# Revisional surgery for persistent dysphagia plus Roux Y gastric bypass robot-assisted in a patient with obesity. About a case

**DOI:** 10.1093/jscr/rjae149

**Published:** 2024-03-17

**Authors:** Santiago A Muñoz-Palomeque, Máximo V Torres Guaicha, Glenda Y Herrera Cevallos, Tábata Lissette Tinoco Ortiz, Amílcar O Herrera Cevallos

**Affiliations:** General and Laparoscopic Surgery, Universidad Internacional del Ecuador, Quito 170411, Ecuador; General Surgery Department, Hospital Metropolitano, Quito 170508, Ecuador; General Surgery Department, Hospital Metropolitano, Quito 170508, Ecuador; Faculty of Medicine, Universidad Central del Ecuador, Quito 170136, Ecuador; General Surgery Department, Hospital Metropolitano, Quito 170508, Ecuador; General Surgery Department, Hospital Metropolitano, Quito 170508, Ecuador; General Surgery Department, Hospital Metropolitano, Quito 170508, Ecuador

**Keywords:** deglutition disorders, bariatric surgery, obesity, fundoplication, robotic surgical procedures

## Abstract

This case study presents a female patient with progressive dysphagia for solids, heartburn, and obesity that proved refractory to clinical management. Imagenological diagnosis revealed esophageal stenosis and achalasia. Furthermore, metabolic syndrome was established. We proposed intervention through esophagogastric reconstruction due to stenosis, revision of cardiomyotomy and robotic gastric bypass revealing scar tissue and fibrosis on the anterior aspect of the stomach resulting from prior fundoplication surgery. The patient underwent esophagogastric reconstruction due to adhesion bands which conditioned partial angulation of the gastroesophageal junction, cardiomyotomy revision, anterior and posterior hiatal plasty, and Roux Y Gastric Bypass assisted by a robot without complications. The intervention resulted in significant improvement in postoperative symptoms. This case highlights the importance of considering the probability of mechanical obstruction due to postsurgical adhesions in the initial evaluation of recurrent and persistent dysphagia, with surgical reintervention being the ideal option for resolution.

## Introduction

Postoperative fundoplication dysphagia is a widespread phenomenon that demands an integral approach for optimal treatment [1]. Severe and persistent dysphagia due to esophageal hiatus stricture is a rare but serious complication, often following antireflux procedures. This pathology negatively impacts the quality of life of affected patients, requiring surgical intervention in ~3% of cases, either through endoscopic dilation or surgery [2].

Contributing factors to persistent dysphagia include tight, slipped, or displaced fundoplication, peptic stricture and preoperative esophageal motility disorders. Although rare, persistent dysphagia can also result from hiatal stenosis following laparoscopic surgery for gastroesophageal reflux disease (GERD) [2]. Another potential complication to persistent dysphagia is adhesion syndrome, arising during tissue repair, forming intraperitoneal adhesions that may impact the quality of the repair [3].

On the other hand, talking about metabolic surgery in contrast to clinical management, procedures such as Roux Y gastric bypass (LRYGB), has shown lower mortality rates and increased life expectancy [4]. The American Society for Metabolic and Bariatric Surgery (ASMBS) recommends metabolic surgery for individuals with a body mass index (BMI) of 30 to 34.9 kg/m^2^ and obesity-related comorbidities, particularly type 2 diabetes mellitus, where substantial weight loss has not been achieved through non-surgical means [5].

## Case report

We report a 47-year-old female patient, with a complex medical history, including esophageal achalasia resolved by cardiomyotomy and laparoscopic fundoplication 27 years prior, grade II obesity and metabolic syndrome, presented now with an 8-year history of progressive dysphagia for solids associated with dyspepsia, vomiting, heartburn and postprandial epigastralgia. These symptoms led to a diet modification in recent months to a high-calorie liquid regime. Despite physical activity and dietary modifications, there was substantial weight gain with difficulty in controlling this increase, resulting in basal blood glucose and blood pressure increase. At the time of evaluation, the patient had a BMI of 36.11 kg/m^2^, abundant abdominal adipose panicle and reported mild epigastric pain at a Visual Analogue Scale 3/10 during the exploration, without signs of peritonism. Paraclinical assessment revealed fasting glucose of 151.12 mg/dl, elevated C-peptide of 3.29 ng/ml, LDL-c of 155 mg/dl, HDL-c of 47.15 mg/dl, and triglycerides of 341 mg/dl. Abdominal ultrasound suggested grade I/II hepatic steatosis and gallbladder polyps measuring 8 and 8.8 mm with a slight amount of biliary sludge.

**Figure 1:**
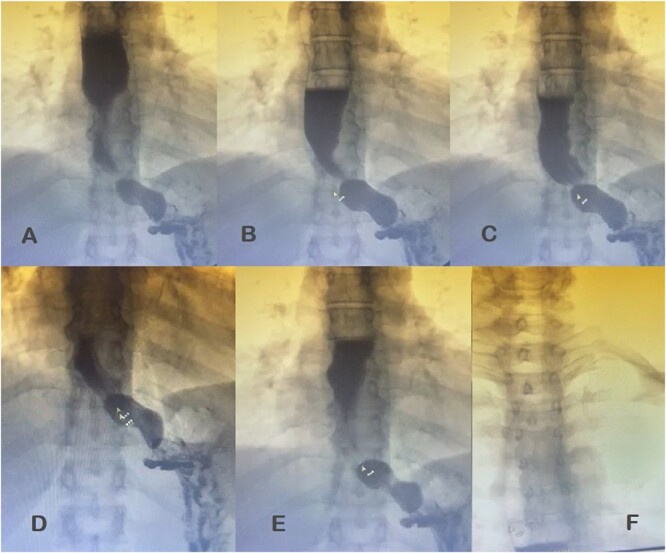
Esophagogastroduodenal transit with barium contrast. (A) Enlarged esophagus; (B) area of stenosis; (C-E) fundoplication and hiatal hernia with stomach above the diaphragmatic domes; tertiary F waves.

**Figure 2:**
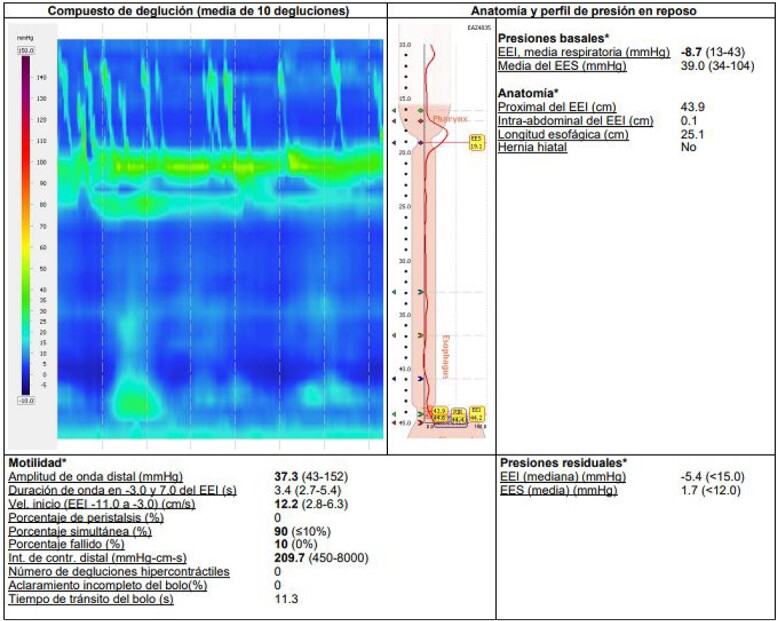
Esophageal manometry with evidence of panpressurization (achalasia type II).

Additional diagnostic procedures included videoendoscopy, which revealed grade II megaesophagus and gastroscope-adjusted hiatus to the retroversion maneuver with signs of competent fundoplication. Esophagogastroduodenal transit with barium contrast (Fig. 1) showed adequate passage from the esophagus to the stomach with fundoplication on diaphragmatic arches, suggesting the presence of a hiatal hernia with intrathoracic stomach contents. Return of contrast after swallowing suggests reflux, presence of tertiary waves, stenotic area in cardia, and megaesophagus without alteration of gastric emptying. The patient showed normal phmetry and manometry (Fig. 2) with difficulty in passing the probe through the lower sphincter, presumably due to hypertension or lack of relaxation and esophageal body activity with panpressurization, classified as achalasia evolving from type I to type II.

**Figure 3:**
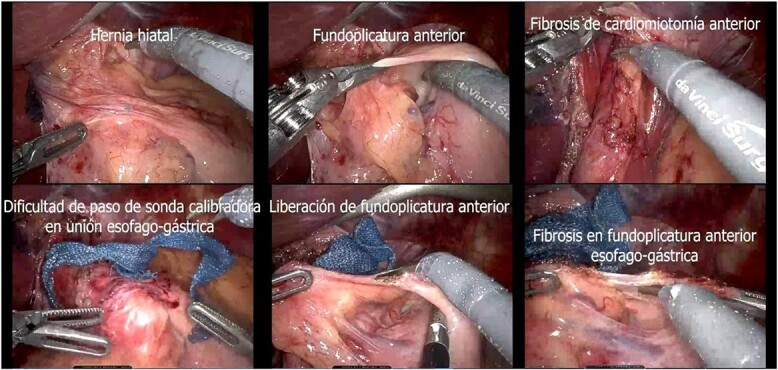
Transsurgical findings (hiatal hernia; anterior funfuplication; fibrosis of anterior cardiomyotomy; difficulty in passing the calibrating probe in the gastroesophageal junction; release of anterior fundoplication; fibrosis in anterior esophagogastric fundoplication).

**Figure 4:**
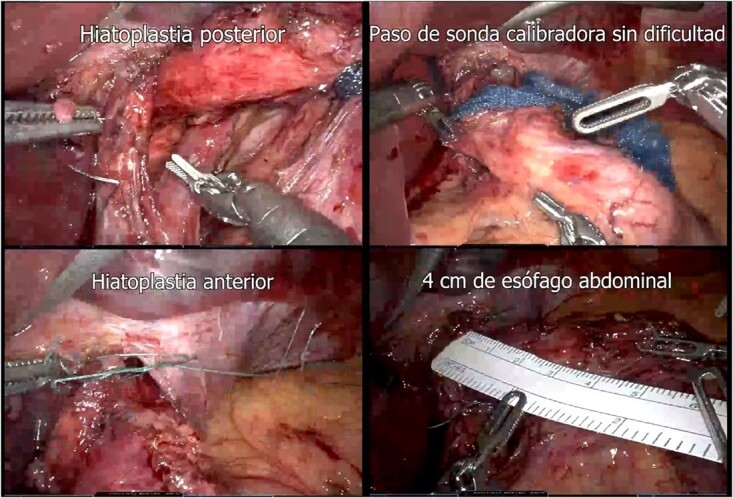
Hiatoplasty (posterior hiatoplasty; passage of gauge probe without difficulty; anterior hiatoplasty; 4 cm of abdominal esophagus).

**Figure 5:**
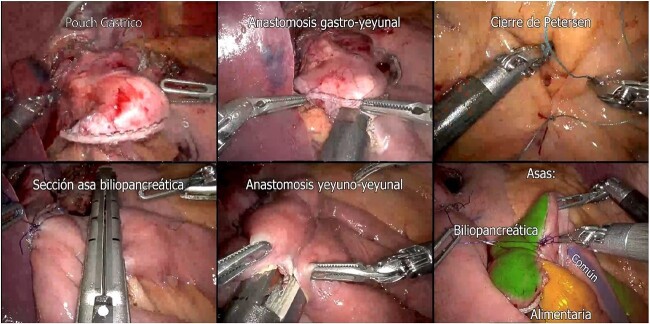
Preparation of Roux-en-Y Gastric Bypass (gastric pouch; gastrojejunal anastomosis; Petersen closure; biliopancreatic loop section; jejunojejunal anastomosis; biliopancreatic, common and alimentary loops).

**Figure 6:**
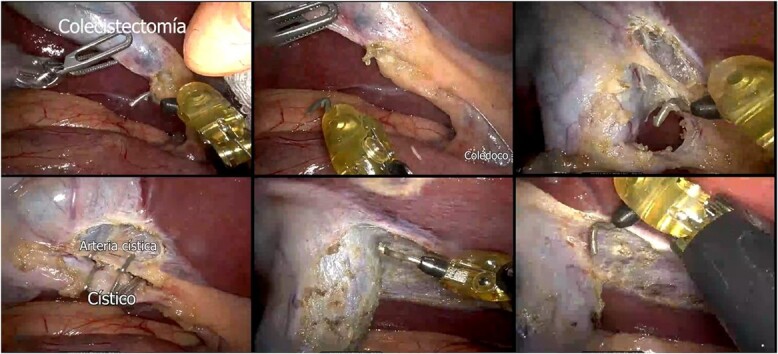
Robot-assisted laparoscopic cholecystectomy.

**Figure 7:**
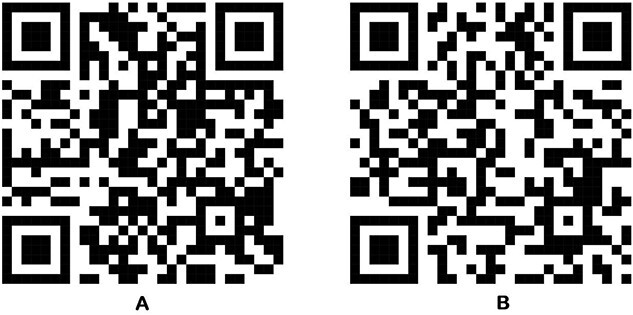
Video links: (A) esophagram; (B) surgical procedure.

A presumptive diagnosis of grade II megaesophagus, esophageal stenosis, achalasia, hiatal hernia with previous fundoplication (Fig. 3), grade II obesity, vesicular polyposis and metabolic syndrome. To resolve these, we performed an esophagogastric reconstruction that accounts for to adhesions, cardiomyotomy revision, anterior and posterior hyatoplasty (Fig. 4), adhesiolysis, LRYGB (Fig. 5), and robotic-assisted laparoscopic cholecystectomy (Fig. 6) without complications. The links for the videos of the esophagogastroduodenal transit and the surgical procedures are available in Fig. 7.

After the procedure, the patient showed favorable postoperative course and was discharged from medical care after 48 hours for follow-up in an outpatient consultation setting.

## Discussion

While the precise etiology of postsurgical dysphagia remains elusive, previous research suggests that preoperative characteristics of the lower esophageal sphincter such as dysphagia and altered motility, as well as surgical technique can influence the development of persistent dysphagia [2].

Throughout the progression of this case, the primary evaluation focused on the patient’s clinical presentation, where intermittent dysphagia with solid foods indicated structural deterioration [2], especially given the patient’s history of fundoplication and myotomy. Notably, medical literature has shown that up to one-fifth of patients that undergo fundoplication surgery may continue to experience persistent dysphagia [2].

The most common causes of recurrent dysphagia include excessive scarring at the distal edge of the myotomy, incorrect fundoplication, effects of previous treatment, GERD, intrathoracic migration, stricture, angulation and esophageal cancer. A comprehensive evaluation of these factors is critical for diagnosis, which can be achieved through scrutiny of surgical history as well as medical procedures such as endoscopy, barium esophageal transit and esophageal manometry [1, 6–8].

One of the challenges of dealing with a short myotomy or distal scar, an area of high pressure might sometimes impose difficulties to cross the gastroesophageal junction, as exemplified in our patient. In such instances the catheter can only be placed in the stomach with the assistance of endoscopic guidance [8].

Preoperative tests often fail to determine the causes of dysphagia with certainty; thus, the definitive diagnosis is typically achieved intraoperatively through early laparoscopy [2]. Following paraclinics analysis using tests such as endoscopy, barium esophageal transit and esophageal manometry, gastroesophageal reconstruction was initially considered to correct the previous myotomy and fundoplication. However, due to the transsurgical findings we opted for adhesions release, obtaining angulation correction of the gastroesophageal junction and demonstrating adequate passage of the caliper. Reoperation clarified the obstructive nature of the dysphagia, identifying excessive scarring and fibrous tissue formation around the hiatus as the actual cause of obstruction and consequent dysphagia.

Given the criteria proposed by the ASMBS [5], additional bariatric intervention for the patient’s refractory obesity was justified. LRYGB was chosen due to its known capacity to resolve reflux and significant long-term effectiveness in weight loss, in addition to the recorded lower prevalence of esophagitis for this procedure compared to the gastric sleeve [4]. Robotic assistance facilitated precision during the procedures, ensuing good post-surgical resolution and early recovery without complications, or the need for strong analgesics or opioids.

## Conclusions

The approach to studying persistent dysphagia must be systematic. Adhesions due to excessive scarring that cause stenosis or angulation of the gastroesophageal junction should be considered within the presumptive diagnoses in dysphagia in patients. Particularly in cases that present a history of surgically resolved achalasia or that have undergone fundoplications. If such diagnosis is confirmed, we recommend to perform new surgery to resolve the mechanical obstruction.

This clinical case highlights the significance of conducting a comprehensive evaluation and a personalized surgical approach for patients experiencing persistent post-fundoplication dysphagia. The integration of metabolic procedures when necessary, and the utilization of high-end technologies such as robotic assistance, are essential components that contribute to enhanced surgical outcomes.
